# Maternal nut and fish consumption during pregnancy and child risky decision-making at 11 years old

**DOI:** 10.1007/s00787-025-02750-5

**Published:** 2025-06-10

**Authors:** Marina Ruiz Rivera, Ariadna Pinar-Martí, Izaro Babarro, Jesús Ibarluzea, Jesús Vioque, Sabrina Llop, Ana Fernández-Somoano, Adonina Tardón, Vicenç Pascual-Rubio, Albert Fabregat-Sanjuan, Silvia Fernández-Barrés, Dora Romaguera, Mònica Guxens, Jordi Julvez

**Affiliations:** 1 Clinical and Epidemiological Neuroscience (NeuroÈpia), Institut d’Investigació Sanitària Pere Virgili (IISPV), Reus, Catalonia 43204 Spain; 2 Barcelona Institute for Global Health (ISGlobal) - Campus MAR, PRBB, Barcelona, Catalonia Spain; 3 Universitat Pompeu Fabra (UPF), Barcelona, Catalonia Spain; 4 Faculty of Psychology, University of the Basque Country (UPV/EHU), Donostia/San Sebastian, 20018 Spain; 5 Group of Environmental Epidemiology and Child Development, Biodonostia Health Research Institute, Donostia/San Sebastian, 20014 Spain; 6 Spanish Consortium for Research on Epidemiology and Public Health (CIBERESP), Instituto de Salud Carlos III, Madrid, 28029 Spain; 7 Sub-Directorate for Public Health and Addictions of Gipuzkoa, Ministry of Health of the Basque Government, Donostia/San Sebastian, 20013 Spain; 8 Instituto de Investigación Sanitaria y Biomédica de Alicante, Universidad Miguel Hernández (ISABIAL-UMH), Alicante, 03010 Spain; 9 Epidemiology and Environmental Health Joint Research Unit, FISABIO-Universitat Jaume I- Universitat de València, Valencia, Spain; 10 IUOPA Public Health Department, University of Oviedo, Asturias, Spain; 11 Institute of Health Research of the Principality of Asturias-Foundation for Biosanitary Research of Asturias (ISPA- FINBA), Oviedo, Asturias Spain; 12 Departament d’Enginyeria Mecànica, Universitat Rovira i Virgili, Tarragona, Spain; 13 Agència de Salut Pública de Barcelona, Barcelona, Spain; 14 Health Research Institute of the Balearic Islands (IdISBa), Palma, Spain; 15 CIBER Physiopathology of Obesity and Nutrition (CIBEROBN), Madrid, Spain; 16 Department of Child and Adolescent Psychiatry/Psychology, Erasmus University Medical Center, Rotterdam, Netherlands; 17 ICREA, Barcelona, Spain; 18 Facultat de Medicina i Ciències de la Salut, Human Nutrition Unit, Universitat Rovira i Virgili, Reus, Spain; 19 NeuroÈpia Group, Institut d’Investigació Sanitària Pere Virgili (IISPV), Hospital Universitari Sant Joan de Reus, 43204 Reus (Tarragona), Catalonia, Spain

**Keywords:** Nuts, Fish, Pregnancy, Impulsivity, Decision-making, Risk, Children

## Abstract

Antenatal diet may have a role in the development of impulsivity, and hence in risky decision-making. This study is assessing whether nut and fish consumption during pregnancy is associated with impulsivity and risky decision-making until pre-adolescence. This is a mother-child population-based birth cohort study, INMA (Infancia y Medio Ambiente) project (recruitment years 2004–2008). The final sample included 1386 healthy preadolescents and their mothers. The exposure variables included maternal nut and fish consumption during the first trimester of pregnancy from a validated food frequency questionnaire (FFQ). The main outcomes were impulsivity index (Attention Network Task, ANT) and the number of risky decision-making (Roulette Task). The association was estimated by multi-variable linear regression models. Children whose mothers were at the highest nut intake tertile showed less risky decision scores compared to those at the lowest tertile (β = -1.49, 95%CI = -2.85; -0.14; p-for-trend = 0.03). Fish consumption showed a positive association with ANT impulsivity index (the coefficient for second quintile compared to the lowest β = 65.73, 95%CI = 1.11; 130.35), with a p-value < 0.05 and a p-for-trend = 0.61. Although, no association was observed between fish consumption and risky decision-making outcome. Our study suggests that a higher nut intake during early pregnancy may be related with less risky decision-making in the 11-year-old children. Also, that a moderate intake of fish during early pregnancy seems to be associated with higher impulsivity in the offspring. The latter finding may be indicative of no benefits observed from seafood consumption.

**Supplementary Information:**

The online version contains supplementary material available at 10.1007/s00787-025-02750-5.

## Implications and contribution

Fish intake during pregnancy has been associated with increased cognitive function scores later in childhood. However, nut intake during pregnancy and child neuropsychological development is barely investigated.

This study adds more evidence of this potential associations with complex cognitive functions like impulsivity and risky decision-making outcomes in preadolescents.

This new evidence with the neuropsychological functions like risky decision making, adds more evidence and social awareness of the importance of pregnancy nutrition for late child mental health and its potential consequences in psychological functioning.

## Introduction

For a long time, impulsivity has been considered a personality trait. According to Kagan’s theory, impulse control was considered a temperament which influenced cognitive and behavioral responses in children [1]. However, it is currently known that impulsive behaviors can be related with the maturation of inhibitory control during adolescence, which is associated with an increased involvement of the prefrontal cortex maturation [2].

Impulsivity could be considered as a predisposition towards risky behaviors [3]. This relationship is partially due to its association with the biological bases of many disorders, such as conduct disorder or substance abuse [4]. Nevertheless, the factors that influence the way we act and the decisions we make are manifold (i.e., school engagement [5, 6], family connectedness and social and physical environment [7, 8]). However, some theories propose that variables within the ecology of human development are inexorably interconnected and may act in cascade. Consequently, impulsivity might play a significant role in mental health, particularly influencing risky decisions that may have consequences in the child psychological development and wellbeing.

According to the World Health Organization (WHO), maintaining a healthy diet during pregnancy is not only critical for mothers’ health but also for health and neurodevelopment of their children [9]. Specifically, it has been found that a suboptimal diet during pregnancy, a stage in which the brain experiences a peak of growth, may be related with long-term cognitive development and behavioral performance [10]. Previous evidence suggests that the primary gestational micronutrient related with children’s brain development is the intake of long-chain polyunsaturated fatty acids (LC-PUFAs), with fish and nuts being their most recognized healthy sources [11, 12]. These LC-PUFAs are essential for the development of the prefrontal cortex, specifically in key regions related to inhibition response and decision-making [2, 13].

Despite the inherent exposure to trace contaminants [14–16], fish consumption during pregnancy is related to improvements in neuropsychological assessments among children, as reported by us and others [10, 17, 18]. This is due in part to the effect of LC-PUFA intake on brain development [19]. Although nuts also contain a considerable amount of PUFAs, there is little evidence about the relation between its maternal consumption and child neuropsychological improvements. In spite of this, a prior study from Gignac et al. found that nut intake during early pregnancy was associated with long-term child neuropsychological development up to 8 years old [20]. Thus, we hypothesize that a diet rich in LC-PUFAs during pregnancy could improve impulse control and decision-making, potentially reducing risky behaviors in children and preadolescents. However, nuts are also rich in polyphenols, minerals and vitamin E, and seafood also contain selenium, iodine and vitamin D. These nutrients may also have beneficial effects to the brain development during pregnancy [17, 20].

Despite these findings, to the best of our knowledge, there is limited literature examining the association between nuts or fish intake and specific neuropsychological functions such as impulsivity and risky behaviors. Studying these functions jointly would bring a more complete perspective and it would help to understand better the role of nutrition during pregnancy in children’s complex behaviors. Thus, the aim of the present study is to assess the association between nut and fish consumption during pregnancy and impulsivity and risky decision-making until preadolescence.

## Methods

### Study population

The participants of this study were families of The Spanish Environment and Childhood (Infancia y Medio Ambiente, INMA) Project. This is a multicenter prospective birth cohort study which included data from participants of cohorts along different regions of Spain (Supplementary Fig. S1). We used data from four population-based birth cohorts (Asturias, Gipuzkoa, Valencia and Sabadell). 2598 women during the first trimester of gestation were recruited in 2004–2008. Finally, 2498 offspring were enrolled at birth. The sample was selected based on data availability: 1386 children were followed up to the 11th year visit with all the required data for this study, including neuropsychological examinations. For the risky decision-making assessment, there were 1081 participants, as this data was not collected for participants in the Valencia cohort. In the final models, we only included those participants with complete data on the covariates, ending up with 1346 participants for impulsivity regression models and 985 for decision-making models. The study protocol was approved by hospital and institutional ethics committees in each region. The informed consent of all participants was collected at recruitment and at each follow-up.

### Exposure and co-variable information

Two main exposures were considered for the present study, both related to the intake of foods naturally rich in PUFAs during pregnancy: Fish and nut consumption. A food frequency questionnaire (FFQ) adapted and validated for pregnant women was used to assess fish and nut consumption at the first and third trimesters (10–13 weeks and 28–32 weeks) of pregnancy [21]. All data regarding fish consumption was selected as one of the exposure variables, including fatty fish, lean fish, shellfish, canned tuna, and other types of seafood. Each seafood item was converted to average weekly (w) intakes in grams (g) and then added to determine the total seafood intake (in g/w). For the second exposure variable, total nut intake (including walnuts, almonds, peanuts, pine seeds, hazelnuts) was collected as a single questionnaire item and converted into g/w. It has been used the same single questionnaire item in a previous publication [20].

Further, structured questionnaires were administered in the first and third trimesters of gestation to obtain information on maternal characteristics, including mother age, education level and socio-economic status based on the residential area and mental health clinical history (depression and anxiety) of the mother, among others. The use of omega-3 supplements were also recorded within the FFQs. Furthermore, based on the FFQ data, the maternal energy intake (kcal) and adherence to Mediterranean diet were estimated. Regarding to the latter, it was measured through the relative Mediterranean diet score, constructed using the consumption of vegetables, fruits, legumes, cereals, meat, dairy products and olive oil [22, 23]. We modified the original score by excluding fish and nuts. Finally, type of delivery was collected after giving birth by the nurse involved in the project.

### Executive functions: neuropsychological assessments

Some neuropsychological tests were implemented during the 11th year visit in order to assess child impulsivity and risky behaviors.

Impulsivity was assessed using the Attention Network Test (ANT) [24, 25]. The ANT is a computer-based test to provide a measure of the efficiency of different functions of attention. It consists in a row of five yellow fish appearing either above or below a fixation point is presented. Participants are invited to “feed” the central fish as quickly as possible by pressing either the right or the left arrow key depending on the direction in which the fish in the middle is pointing while ignoring the flanker fish, which points in either the same (congruent) or opposite (incongruent) direction than the middle fish. The impulsivity index used for this study is measured by subtracting the time response of correct answers minus the time response of wrong answers in the test, expressed in milliseconds (ms). Higher scores indicate higher impulsivity.

The instrument used to measure risky behaviors was the Roulettes Task. It is a gambling task aimed to assess risk preferences under gain and loss conditions separately and it was adapted from the Cups Task [26]. The participant is asked to choose between safe and risky options, in order to gain as much money as possible in the long run. On each trial, two wheels (the “roulettes”) are displayed on the screen, each divided by segments of equal sizes and depicting an amount of money. The participant is asked to choose which wheel to spin. The main outcome is the total number of risky choices. Higher scores indicate higher risky decision-making.

### Statistical analysis

Associations between maternal nut and fish consumption during pregnancy and child neuropsychological outcomes were evaluated using separate multivariable linear regression analyses, these models were applied after assessing the statistical assumptions underlying the use of linear regression models – normal distribution of the outcome continuous variables, residuals independence, homoscedasticity and normal distribution of residuals (data not shown).

Both nut and fish consumption were adjusted for energy intake using the residual method [27]. Nut intake was evaluated as an ordinal variable (in tertiles of weekly grams, with the first tertile as the reference category); fish intake was also evaluated as ordinal but categorized in quintiles (with the first quintile as the reference category). The FFQ shows a low level of precision, since it is an indirect estimate and is subjected to a certain degree of recall bias from the reporter. For this reason, it is recommended to use the consumption variables as categorical instead of in continuous format. The number of categories of each exposure variable was decided based on their distribution in g/week and previous literature [17, 20]. Using a low-precision estimate helps to reduce exposure disparity and retain outliers in closer categories. Seafood intake, widely distributed, allowed for quintiles, while the lower distribution of nut consumption required tertiles. In this Spanish sample with a large seafood intake, each quintile roughly equals one serving, making quintiles more clinically meaningful. For nuts, tertiles best capture intake differences in each category. Outcome variables were evaluated as continuous.

**Fig. 1:**
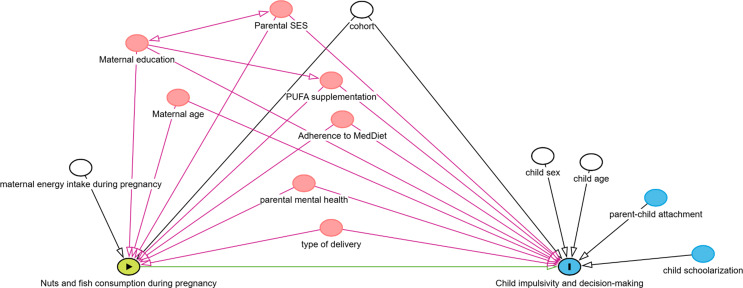
Directed Acyclic Graph model for investigating causal paths between pregnancy nut and fish consumption and child impulsivity and risky decision-making. Minimal sufficient adjustment sets containing child age, child sex, cohort and mother energy intake during pregnancy for estimating the total effect of nuts and fish consumption during pregnancy on impulsivity and decision-making: Maternal age at conception, adherence to Mediterranean diet during pregnancy, PUFAs supplementation, parental socioeconomic status, maternal education, parental mental health and type of delivery. PUFA supplementation was not included in the final models due to a low response rate (2.25%)

Confounders were selected using a Directed Acyclic Graph (DAG) [28] model, as illustrated in Fig. 1, and afterwards with explanatory modeling. Minimally-adjusted regression models included adjustment for sex (male/female) and age of the child (years), cohort, and total maternal energy intake (kcal/day). The fully adjusted models were additionally adjusted for maternal education (primary or less/secondary/university or more), mother age at pregnancy, type of delivery (caesarean/vaginal), anxiety and depression clinical history (yes/no), socio-economic status based on the residential area (in tertiles, from lower to higher deprivation) and adherence to Mediterranean diet (in tertiles, low/medium/high). Omega-3 supplements during pregnancy was not included in the final models due to the low reported prevalence (2.25%). However, its inclusion in the models did not change results (data not shown). Secondary analyses were conducted with the exposure at third trimester of pregnancy. Additionally, as secondary analyses, we checked the characteristics of the study participants and nonparticipants at the 11-year-old period.

Finally, we did not observe any effect change between prenatal seafood and nut intake and cognitive development after adjusting the final models for child seafood and nut intake in secondary analyses from previous INMA studies. We decided to keep present analyses with the main hypothesis focus and not to re-test secondary analyses previously reported [17, 20].

All analyses were conducted with RStudio statistical software package (4.0.4 version), and statistical significance was defined with a p-value < 0.05.

## Results

Baseline characteristics of study participants according to their nut consumption during the first trimester of pregnancy distributed in tertiles are shown in Table 1. Mothers classified in the high nut consumption group presented a higher educational level (41% have completed university studies), had a vaginal delivery (85%), and lived in a less deprived socio-economical area (49%). Mothers who had a higher consumption of nuts also showed a higher adherence to Mediterranean diet (31%) and reported lower energy intake (mean, 1987.73 ± 491.54 kcal/day). The distribution of the outcomes was slightly different between nut intake categories. Similar data descriptions were found examining the baseline characteristics of the participants according to their fish consumption during the first trimester of pregnancy classified into quintiles (Table 2). The children’s age mean during neuropsychological examination was 11.18 years (Standard Deviation (SD), 0.70 years). Finally, the two neuropsychological outcomes exhibited a weak negative correlation (ρ = -0.0878, p-value = 0.0038). We found that the mothers from completed cases had better socio-demographic characteristics than those mothers from incomplete cases at the 11th year follow up (see Supplementary Tables S1 and Supplementary Table S2).

**Table 1:** Baseline characteristics of the study participants according to tertiles of maternal nut consumption in the first trimester of pregnancy

		Tertiles of maternal nut intake			*p*-values
		Low (median = 0.00 g/week)	Medium (median = 17.43 g/week)	High (median = 73.43 g/week)	
		(*n* = 862)	(*n* = 862)	(*n* = 862)	
*Maternal characteristics*					
Age in years, mean, (SD)		30.35 (4.55)	30.59 (4.33)	30.85 (4.23)	0.06
Cohort location, n (%)	Asturias	189 (22)	128 (15)	166 (19)	< 0.01
	Gipuzkoa	165 (19)	171 (20)	291 (34)	
	Sabadell	238 (28)	222 (26)	194 (23)	
	Valencia	270 (31)	341 (40)	211 (24)	
Education, n (%)	Primary school or less	245 (28)	234 (27)	169 (20)	< 0.01
	Secondary school	358 (42)	372 (43)	336 (39)	
	University or more	257 (30)	255 (30)	356 (41)	
Socio-economic level based on residential area, n (%)	Low deprived	308 (36)	331 (39)	417 (49)	< 0.01
	Medium deprived	380 (45)	366 (43)	325 (38)	
	High deprived	159 (19)	148 (18)	111 (13)	
Energy intake in kcals/day during the first trimester of pregnancy, mean (SD)		2026.49 (518.92)	2346.10 (584.51)	1987.73 (491.54)	< 0.01
Fish intake in g/week, mean (SD)		487.13 (292.25)	490.49 (273.07)	496.51 (262.43)	< 0.01
Anxiety up to first trimester, n (%)	Yes	124 (14)	128 (15)	119 (14)	0.83
Depression up to first trimester, n (%)	Yes	82 (10)	102 (12)	73 (8)	0.06
Type of delivery, n (%)	Vaginal	642 (79)	669 (82)	679 (85)	0.01
Adherence to Mediterranean diet during pregnancy, n (%)	Low	362 (42)	384 (45)	327 (38)	
	Medium	279 (32)	287 (33)	267 (31)	< 0.01
	High	221 (26)	191 (22)	268 (31)	
*Child characteristics*					
Impulsivity index, mean (SD)		300.61 (369.08)	222.77 (384.40)	248.92 (355.04)	0.01
Number risky choices, mean (SD)		30.01 (8.82)	29.79 (9.57)	29.07 (8.94)	0.35
Sex, n (%)	Female	389 (47)	407 (49)	407 (49)	0.56

SD: Standard deviation

^a^ Some of the totals do not match the total number of subjects due to missing values in some categories

^b^ ANOVA p-values for continuous variables and chi-square p-value for the categorical one

**Table 2:** Baseline characteristics of the study participants according to quintiles of maternal fish consumption in the first trimester of pregnancy

			Quintiles of maternal fish intake					*p*-values
			Q1 (median = 189.84 g/week)	Q2 (median = 330.26 g/week)	Q3 (median = 446.46 g/week)	Q4 (median = 585.97 g/week)	Q5 (median = 844.76 g/week)	
			(*n* = 518)	(*n* = 517)	(*n* = 517)	(*n* = 517)	(*n* = 517)	
*Maternal characteristics*								
Age in years, mean (SD)			29.32 (4.8)	30.46 (4.48)	30.75 (4.06)	30.98 (4.08)	31.47 (4.11)	< 0.01
Cohort location, n (%)	Asturias		83 (16)	75 (15)	87 (17)	145 (28)	100 (19)	
	Gipuzkoa		77 (15)	118 (23)	137 (27)	128 (25)	202 (39)	< 0.01
	Sabadell		122 (24)	131 (25)	125 (24)	127 (25)	101 (20)	
	Valencia		236 (45)	193 (37)	168 (33)	117 (23)	114 (22)	
Education, n (%)	Primary school or less		184 (36)	146 (28)	114 (22)	112 (22)	92 (18)	< 0.01
	Secondary school		200 (39)	210 (41)	203 (39)	214 (41)	239 (46)	
	University or more		133 (26)	161 (31)	198 (38)	191 (37)	185 (36)	
Socio-economic level based on residential area, n (%)	Low deprived		161 (32)	222 (44)	231 (46)	236 (46)	206 (40)	
	Medium deprived		237 (47)	213 (42)	195 (39)	210 (41)	216 (42)	< 0.01
	High deprived		105 (21)	73 (14)	80 (16)	68 (13)	92 (18)	
Energy intake in kcals/day during the first trimester of pregnancy, mean (SD)			2211.22 (613.07)	2134.01 (538.96)	2137.93 (597.60)	2044.60 (502.88)	2072.61 (507.82)	< 0.01
Nut intake in g/week mean (SD)			38.29 (66.64)	39.83 (76.58)	38.15 (59.5)	49.98 (78.96)	40.67 (84.07)	< 0.01
Anxiety up to first trimester, n (%)	Yes		80 (15)	72 (14)	75 (14)	69 (13)	75 (14)	0.91
Depression up to first trimester, n (%)	Yes		68 (13)	55 (11)	43 (8)	45 (9)	46 (9)	0.06
Type of delivery, n (%)	Vaginal		380 (79)	392 (80)	408 (85)	410 (84)	400 (81)	< 0.01
Adherence to Mediterranean diet during pregnancy, n (%)	Low		283 (55)	235 (46)	220 (43)	178 (34)	157 (30)	< 0.01
	Medium		134 (26)	160 (31)	162 (31)	181 (35)	196 (38)	
	High		101 (20)	122 (24)	135 (26)	158 (31)	164 (32)	
*Child characteristics*								
Impulsivity index, mean (SD)			227.13 (389.65)	285.61 (368.73)	263.05 (362.83)	244.04 (340.15)	263.92 (388.81)	0.53
Number risky choices, mean (SD)			30.00 (9.18)	30.40 (9.01)	29.11 (9.36)	29.56 (9.28)	29.16 (8.7)	0.76
Sex, n (%)								
	Female		239 (49)	255 (51)	229 (47)	245 (49)	235 (47)	0.68

SD: Standard deviation

a. Some of the totals do not match the total number of subjects due to missing values in some categories

b. ANOVA p-values for continuous variables and chi-square p-value for the categorical ones

Tables 3 and 4 show minimally and fully adjusted associations between nut and fish intake during the first trimester of pregnancy and the neuropsychological assessments, respectively. Regarding nuts (Table 3), no significant association was found with ANT impulsivity index. However, a negative association with risky decision-making score was observed in both minimally and fully adjusted models. Compared to the lowest tertile, those at the highest tertile showed a significantly lower number of risky decisions (β= -1.53, 95% CI = -2.87; -0.20, *p* < 0.05; and β= -1.49, 95% CI = -2.85; -0.14, *p* < 0.05, for minimally and fully adjusted models, respectively). Further, a significant linear trend across nut intake tertiles was observed in both minimally and fully adjusted models (*p* for trend = 0.03).

**Table 3:** Multivariable linear regression analysis between nut consumption during the first trimester of pregnancy (divided into tertiles) and child scores at impulsivity and risky decision-making at 11 years old

Neuropsychological outcome		Difference in child’s neuropsychological score						
	Maternal nut intake (1st trim)^a^	Minimally adjusted^b^				Fully adjusted^c^		
		n	β	95% CI	p for trend	β	95% CI	p for trend
ANT - Impulsivity index^d^	Lowest tertile	426	Ref.		0.24	Ref.		0.21
	Middle tertile	436	-46.17	(-96.20, 3.87)		-45.89	(-96.28, 4.50)	
	Highest tertile	484	-29.22	(-76.61, 18.17)		-31.78	(-79.71, 16.14)	
Cups Task Risky decision-making (number of risky choices)^d^	Lowest tertile	320	Ref.		0.03	Ref.		0.03
	Middle tertile	289	-0.98	(-2.45, 0.50)		-0.84	(-2.33, 0.65)	
	Highest tertile	376	-1.53	(-2.87, -0.20)*		-1.49	(-2.85, -0.14)*	

ANT: Attentional Network Test; CI = Confidence Interval

* Statistically significant (p-value < 0.05)

^a^ Median of nut intake within tertile categories (T1 = 0.00 g/week; T2 = 17.43 g/week; T3 = 73.43 g/week)

^b^ Beta coeficients and 95% CI estimated using linear regression models adjusted by cohort origin, sex and age of the child and kcals intake during pregnancy

^c^ Beta coeficients and 95% CI estimated using linear regression models additionally adjusted by type of delivery (vaginal/caesarean), adherence to Mediterranean diet (excluding fish and nuts) (low/medium/high), maternal educational level (up to primary/secondary/university or more) and age, area-level socioeconomic status based on deprivation index, and maternal mental health clinical history

^d^ Higher scores indicate lower performance of the test

**Table 4:** Multivariable linear regression analysis between fish consumption during the first trimester of pregnancy (divided into quintiles) and child scores at impulsivity and risky decision-making at 11 years old

Neuropsychological outcome		Difference in child’s neuropsychological score						
	Maternal fish intake (1st trim)^a^	Minimally adjusted^b^				Fully adjusted^c^		
		n	β	95% CI	p for trend	β	95% CI	p for trend
ANT - Impulsivity Index^d^	Q1	245	Ref.		0.68	Ref.		0.61
	Q2	251	71.16	(7.23, 135.08)*		65.73	(1.11, 130.35)*	
	Q3	264	64.38	(0.94, 127.81)*		61.17	(-2.90, 125.24)	
	Q4	288	39.47	(-23.17, 102.12)		38.85	(-24.50, 102.19)	
	Q5	298	34.45	(-27.82, 96.74)		35.95	(-27.28, 99.18)	
Cups Task - Risky decision-making (number of risky choices)^d^	Q1	143	Ref.		0.37	Ref.		0.56
	Q2	172	0.19	(-1.80, 2.17)		0.26	(-1.74, 2.27)	
	Q3	194	-1.41	(-3.34, 0.53)		-1.30	(-3.26, 0.65)	
	Q4	236	-0.73	(-2.59, 1.14)		-0.53	(-2.41, 1.35)	
	Q5	240	-0.59	(-2.45, 1.26)		-0.34	(-2.22, 1.55)	

ANT: Attentional Network Test; CI = Confidence Interval

* Statistically significant (p-value < 0.05)

^a^ Median of total fish intake within quintile categories (Q1 = 189.84 g/week; Q2 = 330.26 g/week; Q3 = 446.46 g/week; Q4 = 585.97 g/week; Q5 = 844.76 g/week)

^b^ Beta coeficients and 95% CI estimated using linear regression models adjusted by cohort origin, sex and age of the child and kcals intake during pregnancy

^c^ Beta coeficients and 95% CI estimated using linear regression models additionally adjusted by type of delivery (vaginal/caesarean), adherence to Mediterranean diet (excluding fish and nuts) (low/medium/high), maternal educational level (up to primary/secondary/university or more) and age, area-level socioeconomic status based on deprivation index, and maternal mental health clinical history

^d^ Higher scores indicate lower performance of the test

In Table 4, a positive significant association between fish consumption and ANT impulsivity index was found, both at minimally (β = 71.16, 95% CI = 7.23; 135.08, *p* < 0.05, for the second quintile compared to the lowest quintile) and fully adjusted models (β = 65.73, 95% CI = 1.11; 130.35, *p* < 0.05 for the second quintile). In this case, a significative linear trend across tertiles was not observed in any model.

Generally, results almost did not change between minimally and fully adjusted regression models. The same final models repeated with the exposures during the third trimester of pregnancy showed weaker and none-significant results (data not shown).

## Discussion

In this longitudinal study, we found that higher maternal intake of nuts in the first trimester of pregnancy was not associated with child impulsivity index, but it was associated with taking less risky decisions in their children 11 years later. Indeed, they showed a significant protective trend by maternal nut consumption in tertile groups. Both behaviors are closely interconnected, being risky decision behavior a more complex neuropsychological outcome [3, 4]. In contrast to the previous findings, moderate fish intake during early pregnancy was only associated with higher impulsivity index in the offspring at the same age. The coefficients of this index decreased throughout the fish consumption quintiles. However, the associations with the higher consumption categories were not statistically significant, and no association trend was found. This weak association pattern with fish intake showed an inverse U tendency from adverse to null results.

Results regarding nuts are in accordance with the scarce scientific literature, as previous research found an association between the consumption of nuts and neuropsychological improvements. Concretely, a previous study conducted on INMA population from Gignac et al. suggested that the intake of nuts during pregnancy could be related to an improvement in general cognition up to 8 years old [20]. Further, a study from Pinar-Martí et al. showed that teenagers that increased their nut (walnuts) consumption during 6 months improved their sustained attention, Attention Deficit Hyperactivity Disorder (ADHD) symptoms and fluid intelligence [29]. Although literature related to nut consumption and child neurodevelopment is infrequent, these findings highlight the importance of nut consumption during early stages of life. The current study focuses on specific neuropsychological functions that might be related to the aforementioned findings, since impulsivity could be a component of sustained attention measurements and is also a symptom of ADHD.

The present study findings with nut consumption, could be explained by the fact that nuts contain a considerable amount of LC-PUFAs, which in turn, contributes to the development of neocortical areas involved in impulse control [10, 13]. Risky decision-making, as previously explained, is a complex cognitive function related with impulsivity, and it is particularly important due to its emotional and behavioral processing components, which may play a key role in the development of conduct problems as well. Hence, the fact that a higher nut consumption during pregnancy may result in a less risky decision-making behavior later in the child life can be sustained by this hypothetical pathway.

On the contrary, the associations found with fish intake in this study are not consistent with previous findings. A study conducted by Hibbeln et al. found that limiting weekly seafood consumption to less than 340 g may show adverse associations with early childhood neurodevelopment [15]. Also, Julvez et al. concluded that consumption of total fish during pregnancy presents moderate child neuropsychological benefits, including improvements in cognitive functioning [17]. Nevertheless, the difference between these findings and the present study is that they were focused on general neurodevelopment and in early stages of childhood. In our study, a moderate maternal fish consumption in early pregnancy was associated with a higher impulsivity index in their offspring. As mentioned above, fish is another food naturally rich in LC-PUFAs, so we would expect a protective association. A possible explanation could be the presence of pesticides, mercury, lead, and other kind of heavy metals in fish. Some studies suggest that there is a positive correlation between the exposure to these harmful substances and neurocognitive function, specifically with attention and impulsivity [30, 31]. Indeed, in our findings, exposure to trace contaminants in fish may also affect the association. However, in a previous INMA study, we did not find any confounding effect after adjusting the models for cord mercury levels [17]. Hence, the balance between contaminant toxicity and the positive effects of fish consumption during pregnancy warrants further research.

Alternatively, the weak and inconsistent associations between nut and fish intake and these complex neuropsychological outcomes could be attributed to the age of the participants. Since they are in an early stage of adolescence, their prefrontal cortex is not fully developed and they do not have total control of their lives. As a result, the effects of these dietary exposures might not be observed until later stages of life [2].

Investigating the long-term association of diet during pregnancy on neuropsychological complex functions from a public health point of view is important as it has major implications for public health practice and policy development [10]. Of note, that one single study is not enough to influence and change future nutritional guidelines. But some previous studies have already shown similar benefits with conventional cognitive outcomes as well [17, 20]. Nevertheless, in the hypothetic scenario that after some more additional research, preventive actions finally take place, it could be implemented at early stages of life, before cognitive and behavioral problems emerge, since it may be more feasible and effective to apply nutrition-based preventive measures during pregnancy rather than relying on psychosocial interventions later in life. Further, this study offers insights into the relationship between foods naturally rich in PUFAs from both animal and plant sources, and research regarding nut intake and its association with cognitive performance is still limited [20, 29]. Thus, it provides evidence supporting the importance of adequate neurodevelopment and the prevention of certain conduct problems through different nutrient sources, considering their respective advantages and disadvantages. The new scientific finding here is the inclusion of new behavioral domains such as impulsivity and decision-making, which may be influenced by nut and fish intake during pregnancy due to their nutritional composition [32]. These behavioral domains are crucial because of their significant psychosocial consequences, making them essential to measure and incorporate in more neuro-epidemiological studies in order to promote a fully and healthy neurodevelopment. Lastly, the number of subjects selected to participate in this study is relatively large and they have been followed all life since an antenatal stage.

However, this study also faced some limitations. First, data is only available up to 11 years of age. At this stage, the prefrontal cortex is beginning to mature but is not yet fully developed. [33] It would be interesting to conduct the same study with subjects in later adolescence, a stage in which the prefrontal cortex has grown significantly [2]. Second, given the significant time gap between exposure and outcome, there may be many confounders influencing our results that have not been accounted for. Thus, despite the longitudinal nature of this study, a definitive causal relationship cannot be established. Additional studies or randomized controlled trials would be needed to establish stronger proof of causality. Third, there is an unavoidable measurement error in dietary assessments because they are self-reported, likely leading to error in the effect size [34]. Fourth, the generalizability of the findings is limited to one country, Spain, where mothers report high levels of seafood and nut consumption. It would be advisable to replicate this study with other population-based birth cohort samples from other regions of the world that have different levels of nut and seafood consumption. Finally, we cannot rule out the possibility of some degree for selection bias due to attrition during follow up, since the study participants of the completed cases at the 11th year visit, showed improved socio-demographic characteristics compared with nonparticipants.

Overall, the present study suggests that nut consumption during the first trimester of pregnancy might be a protective factor against risky decision-making, while moderate fish consumption during the same stage might act as a risk factor. Further studies are necessary to investigate the consistency of our findings over time, such as applying repeated measurements of impulsivity and risky decision behaviors at older child ages, and including other conduct problem assessments. This new line of research, focusing on specific cognitive domains that develop in late childhood and adolescence, could help to improve future dietary guidelines for pregnant women aiming to prevent risky or problematic behaviors among their offspring.

## Electronic supplementary material

Below is the link to the electronic supplementary material.

Supplementary Material 1

## Data Availability

No datasets were generated or analysed during the current study.

## Abbreviations

ADHD: Attention deficit hyperactivity disorder
ANT: Attention network task
DAG: Directed acyclic graph
FFQ: Food frequency questionnaire
INMA: Infancia y medio ambiente
LC-PUFAs: Long-chain polyunsaturated fatty acids
PUFAs: Polyunsaturated fatty acids
WHO: World health organizations
