# Supplementary material for: Maternal nut and fish consumption during pregnancy and child risky decision-making at 11 years old
Source: Eur Child Adolesc Psychiatry. 2025 Jun 10;34(11):3643–54. doi: 10.1007/s00787-025-02750-5 (PMC12647295; doi:10.1007/s00787-025-02750-5)
Supplement: Supplementary file 1 — Supplementary Material 1 [file 787_2025_2750_MOESM1_ESM.docx]

Supplementary Figure 1. Main phases of the study and flowchart of the population. Spanish Childhood and Environment (INMA) Project, 2004–2020.

Women at first trimester of pregnancy with diet data
*n* = 2586 (98.5%)

Newborns at delivery
*n* = 2498 (94.7%)

40 subjects excluded:
23 Miscarriages
1 Withdrew
16 Lost

Women recruited in the study
N = 2644

*Asturias n = 493
Gipuzkoa n = 630
Sabadell n = 656
Valencia n = 847*


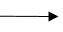


88 subjects excluded:
37 Miscarriages
5 Fetal deaths
45 Withdrew
1 Lost

Children with at least one neuropsychological test at 11 years old
*n* = 1386 (52.7%)

Women included in the study
 n = 2626

18 subjects excluded because of having babies with particular pathologies

Supplementary Table 1. Sociodemographic characteristics of the study participants* and nonparticipants at the 11-year-old period (ANT-Impulsivity index)

| Impulsivity |  | Complete cases*  (n = 1346) | Incomplete cases  (n = 1419) | p-values |
| --- | --- | --- | --- | --- |
| *Maternal characteristics* |  |  |  |  |
| Age in years, mean, (SD) |  | 31.11 (3.94) | 30.02 (4.80) | <0.01 |
| Cohort location, n (%) | Asturias | 219 (16.27) | 276 (19.45) | <0.01 |
|  | Gipuzkoa | 357 (26.52) | 281 (19.80) |  |
|  | Sabadell | 417 (30.98) | 360 (25.37) |  |
|  | Valencia | 353 (26.23) | 502 (35.38) |  |
|  |  |  |  |  |
| Education, n (%) | Primary school or less | 258 (19.17) | 405 (30.78) | <0.01 |
|  | Secondary school | 550 (40.86) | 557 (42.33) |  |
|  | University or more | 538 (39.97) | 354 (26.90) |  |
|  |  |  |  |  |
| Socio-economic level based on residential area, n (%) | Low deprived | 465 (38.11) | 603 (44.80) | <0.01 |
|  | Medium deprived | 537 (44.02) | 541 (40.19) |  |
|  | High deprived | 218 (17.87) | 202 (15.01) |  |
|  |  |  |  |  |
| Energy intake in kcals/day during the first trimester of pregnancy, mean (SD) |  | 2063.51 (510.10) | 2181.55 (597.10) | <0.01 |
| Nuts intake in g/week, mean (SD) |  | 6.48 (11.55) | 5.29 (9.28) | <0.01 |
| Fish intake in g/week, mean (SD) |  | 73.05 (40.28) | 67.10 (38.30) | <0.01 |
| Anxiety up to first trimester, n (%) | Yes | 172 (12.78) | 210 (15.18) | 0.07 |
| Depression up to first trimester, n (%) | Yes | 124 (9.21) | 143 (10.34) | 0.32 |
| Type of delivery, n (%) | Vaginal | 1112 (82.62) | 895 (81.07) | 0.32 |
| Adherence to Mediterranean diet during pregnancy, n (%) | Low | 517 (38.41) | 564 (44.73) | 0.01 |
|  | Medium | 454 (33.73) | 384 (30.45) |  |
|  | High | 375 (27.86) | 313 (24.82) |  |
| *Child characteristics* |  |  |  |  |
| Sex, n (%) | Female | 680 (50.52) | 533 (46.03) | 0.03 |

Supplementary Table 2. Sociodemographic characteristics of the study participants* and nonparticipants at the 11-year-old period (Cups Task -Risky decision choices)

| Cups |  | Complete cases*  (n = 985) | Incomplete cases  (n = 1780) | p-values |
| --- | --- | --- | --- | --- |
| *Maternal characteristics* |  |  |  |  |
| Age in years, mean, (SD) |  | 31.30 (3.86) | 30.13 (4.68) | <0.01 |
| Cohort location, n (%) | Asturias | 219 (22.23) | 276 (15.51) | <0.01 |
|  | Gipuzkoa | 357 (36.24) | 281 (15.79) |  |
|  | Sabadell | 409 (41.52) | 368 (20.67) |  |
|  | Valencia | 0 (0) | 855 (48.03) |  |
|  |  |  |  |  |
| Education, n (%) | Primary school or less | 164 (16.65) | 499 (29.76) | <0.01 |
|  | Secondary school | 402 (40.81) | 705 (42.04) |  |
|  | University or more | 419 (42.54) | 473 (28.21) |  |
|  |  |  |  |  |
| Socio-economic level based on residential area, n (%) | Low deprived | 514 (52.18) | 554 (35.04) | <0.01 |
|  | Medium deprived | 331 (33.60) | 747 (47.25) |  |
|  | High deprived | 140 (14.21) | 280 (17.71) |  |
|  |  |  |  |  |
| Energy intake in kcals/day during the first trimester of pregnancy, mean (SD) |  | 2010.06 (495.87) | 2187.82 (580.68) | <0.01 |
| Nuts intake in g/week, mean (SD) |  | 7.09 (12.67) | 5.19 (8.90) | <0.01 |
| Fish intake in g/week, mean (SD) |  | 77.40 (41.68) | 65.77 (37.34) | <0.01 |
| Anxiety up to first trimester, n (%) | Yes | 117 (11.88) | 265 (15.19) | 0.02 |
| Depression up to first trimester, n (%) | Yes | 89 (9.04) | 178 (10.21) | 0.32 |
| Type of delivery, n (%) | Vaginal | 838 (85.08) | 1169 (79.80) | <0.01 |
| Adherence to Mediterranean diet during pregnancy, n (%) | Low | 343 (34.82) | 738 (45.50) | <0.01 |
|  | Medium | 341 (34.62) | 497 (30.64) |  |
|  | High | 301 (30.56) | 387 (23.86) |  |
| *Child characteristics* |  |  |  |  |
| Sex, n (%) | Female | 487 (49.44) | 726 (47.79) | 0.42 |
|  |  |  |  |  |
